# Looking back to look forward: Learning from past innovations in family medicine training

**DOI:** 10.4102/safp.v68i2.6249

**Published:** 2026-01-23

**Authors:** Ian Couper, Jannie Hugo, Julia Blitz, Hoffie Conradie

**Affiliations:** 1 Division of Rural Health (Ukwanda), Faculty of Medicine and Health Sciences, Stellenbosch University, Cape Town, South Africa; 2 Department of Family Medicine, Faculty of Health Sciences, University of Pretoria, Pretoria, South Africa; 3 Department of Health Professions Education, Faculty of Medicine and Health Sciences, Stellenbosch University, Cape Town, South Africa; 4 Division of Family Medicine and Primary Care, Faculty of Medicine and Health Sciences, Stellenbosch University, Cape Town, South Africa

**Keywords:** family medicine training, medical education, pedagogy, learning, innovation, postgraduate programmes

## Abstract

Under the leadership of the late Professor Sam Fehrsen, the Medunsa MPraxMed, which commenced in 1979, was an innovative programme offering doctors in South Africa the unique opportunity to be trained in family medicine within the context of their workplaces. This article describes its pedagogical approach that profoundly influenced a generation of family physicians. Eight shifts in learning arose out of the programme’s development and iterative renewal. These included a focus on context and real-world application, greater autonomy for students with learning being focused on their needs, modelling the doctor–patient relationship in the educator-student relationship, facilitation of learning more than teaching, offering resources rather than courses, lecturers and students being co-learners and supporting reflective action. Most of the shifts in the Medunsa MPraxMed are now recognised as good educational practice underpinned by evidence, and are still necessary today.

**Contribution:**

Current postgraduate family medicine training programmes in South Africa and beyond are offered the opportunity to reflect on what they might learn from the past approaches of the Medunsa programme.

## Introduction

As we reflect on how we can do better in our educational practices, we recognise how much being part of an innovative family medicine training programme more than 25 years ago continues to inform our pedagogical approaches. That programme was ahead of its time and developed principles that we continue to use today. The purpose of this article is to share that experience with a current readership, reflecting on its ongoing value today.

The Masters in Family Medicine (MPraxMed) course at the Medical University of Southern Africa (Medunsa), now called the Sefako Makgatho University of Health Sciences, was established in 1979 by the late Professor Sam Fehrsen, one of the early pioneers in establishing Family Medicine in South Africa and past editor of the *SA Family Practice Journal*.^1^ The aim from the outset was to make the programme relevant to practising clinicians, particularly those working in underserved areas. As a result, the course was offered to, and largely attracted, students distant from the campus, leading to regionalisation of much of the training with support from part-time facilitators closer to students’ contexts; the four authors all worked in that capacity on the programme in the 1990s.

Working doctors would enrol for between 3 and 6 years of part-time study to complete the course. Over 140 family physicians graduated from the programme by the time Professor Sam Fehrsen retired in 1999. The majority of these graduates were practising in rural and remote areas and many of these later became leaders in family medicine across Southern Africa and beyond.

The Medunsa MPraxMed was regularly renewed, responding to student evaluations and the needs of people in rural areas. Changes coalesced around eight shifts in the way learning might best be facilitated (see Table 1). Rather than describing the theoretical basis of these approaches here, we share our experience of implementing these principles.

**TABLE 1:** The eight shifts of learning facilitation.

Shift	From	Towards
1	The University context	Students’ practice context
2	Teaching	Self-initiated learning
3	Academic control	Facilitation of learning
4	Fixed courses	Providing resources
5	Speaking	Listening
6	Posing as experts	Becoming co-learners
7	Being assessment-driven	Being relevant to student and community needs
8	Theoretical knowledge	Reflective action

## The eight shifts

### From the university campus context towards the students’ context of practice in communities

From the outset, there was a clear understanding of the importance of the context of learning, which meant that the focus shifted from the university campus to the practice locality of the doctors. While students were required to attend a 5-day course annually at the university, the focus was on the context of the students who were located all over South Africa, mainly in rural hospitals, districts and practices. However, much of the learning happened in the practice context, supported by regional seminars to help students reflect on and change their practice, and to build local networks. In recent times, decentralised or distributed medical education has become very common,^2,3,4^ and the importance of context has also been emphasised.^5,6,7^ Yet, Fehrsen was standing on the shoulders of others in grounding the MPraxMed in the context of his trainees. Freire had challenged the way that educational institutions colonise thinking so that students come to believe that their lived experiences in context are irrelevant to learning, whereas these are the basis of critical consciousness and action.^8^ Fehrsen would certainly have agreed with Gruenewald, who proposed a critical pedagogy of place, which challenges educational practices that disregard place,^9^ to focus on more socially and ecologically just approaches. Reid, a graduate of the Medunsa MPraxMed, has argued that this provides a theoretical framework for a distinct rural pedagogy.^10^

For many years, there was a stream for full-time family medicine registrars doing the MPraxMed who were located within the teaching hospital complex, but this was phased out because the context of learning was too far removed from primary care in the periphery. Although this was commonly how family medicine training was provided internationally, Fehrsen came to believe that it was difficult for students to learn the principles and practice of family medicine when working in specialised departments or sub-departments in large, central hospitals, where the whole ethos is biomedical, fragmented and disease-centred rather than biopsychosocial, continuous, patient-centred and community-based. This does not mean that specialists did not, and do not, have a role to play in teaching appropriate skills, but they should not control what, where and how Family Medicine is taught.

Here is an example of how this shift influenced the course. In 1997, doctors from three rural hospitals in one province, all generalists, enrolled *en masse* for the course. Obviously, they could not all leave their hospitals at once. Thus, the first 5-day course was held in one of these hospitals, about a 6 hour drive from Medunsa; at the first session, there were 18 MFamMed students as well as non-student doctors from neighbouring areas. An anaesthetist and an obstetrician accompanied the Medunsa team. A lot of learning took place in the wards and theatre – the hospital kept on working while the course was happening, with everyday problems providing learning material. The then medical superintendent of this remote rural hospital wrote:

Just a few weeks ago I could see no future for our Doctors in [X hospital]. Instead now, thanks to the last week events, I can see a bright light and a fascinating path in front of us, and the smile on the faces of all the Doctors, of the Nurses, of the patients, of the Staff in general, tells me that this is just the beginning of a new era for our Hospital and for the Rural areas in general. (Personal communication)

This method went on to be repeated in other regions.

The foregoing example helps to make the point that the value of this shift lies not only in the learning that takes place, but perhaps even more in the value given to the context, validating students’ practice sites as places for everyone’s learning and growing.

### From teaching towards self-initiated learning

Learning from one another and not merely from teachers was seen to be essential. Teachers like to teach. However, what is taught is not necessarily learnt, and certainly not often applied, as we know very well from patient care. Thus, the focus was shifted to learning: how and why do we learn, and how can educators help learning to occur. An important part of the MPraxMed course involved helping students to reflect on their learning process. Education literature now commonly refers to self-regulated learning (SRL)^11^ and self-directed learning (SDL),^12^ both of which include the learner’s active engagement in goal setting and choice of learning strategies as well as the key role of reflection, and which are often used interchangeably.^13,14,15^ At the time we^*^ used the term self-initiated learning, which can be seen to fall under SDL,^16^ because we focused on the motivation that was expected from our students – the course depended on their learning initiative, because we came to realise that if doctors rely upon teachers, they would be stranded as soon as they finished the course, whereas the aim was to provide skills for ongoing learning and adaptability in ever-changing situations.

Furthermore, we recognised that students learn much from each other, and are often more open to learning from peers than from us, because they feel their peers know exactly what they feel and face every day. Peer (and near-peer) learning in clinical education is increasingly common and has been shown to be beneficial.^17^ Clinicians learn from each other all the time, so there were few challenges experienced in using this approach. During training sessions, students in all 3 years of study were mixed during whole group sessions as well as small group discussions, to assist with this learning process. It was an important affirmation for MPraxMed students to discover how much they actually do know, especially in relation to the rural contexts they work in, and also how much they can learn from each other.

### From academic control towards facilitation of a participatory learning process

Historically, learning opportunities were determined by what ‘the experts’ thought students needed to know. In the MPraxMed programme, we sought to let the students identify their own learning needs. As part of that, we became uncomfortable with the term ‘teacher’ or ‘lecturer’, and moved to becoming facilitators of learning. The concept of facilitation of learning in health professions education has mainly been described in the context of nursing education, where it has been argued that students need to develop confidence in their knowledge and skills to translate these into effective practices, which requires them to explore and question rather than passively accepting and modelling, an approach that emphasises the experience of the learner and their individual needs.^18^ This describes well the approach that we adopted, as we strove to hand over control to our students, to let them express their learning needs and agendas, and to work with them in seeking to meet those needs. As part of this, we too sought to move towards being better facilitators, giving feedback to each other and seeking it from our students.

This required individual and group negotiation, but ultimately, it was our intention that every training session should be co-created with students. Students chose their own topics for module assignments, according to the problems they encountered in their practices, and these were assessed on the degree to which the students’ own learning needs were met. For specific year group activities, students in a particular year planned, according to the curriculum guidelines, which themes they wanted to tackle together and in what way.

In small group sessions, the role of the facilitator was to ensure the learning process was as effective as possible, giving input as a fellow group member. This could be frustrating, and the urge to intervene in order to teach was often great, but we learned to trust the group process. One experience was starting off with a new first year group, whose learning process consisted of laboriously reading to the others sections from pre-readings which their colleagues had also read, which left the facilitator (IC) feeling quite desperate! But after reflecting on a couple of sessions such as this, the group itself began to say, ‘Something is wrong; this is not very interesting or educational. How can we do things differently?’ Slowly, the process transformed so that readings were summarised and applied by group members, and fruitful discussions were held on their relevance and applicability. This also helped to develop critical reading skills.

The pull to control was so strong that we failed at times, especially with students who had previously learnt in a system that encouraged spoon-feeding and rote learning. It was particularly difficult when students did not respond to our attempts to share control and found it difficult to participate in this new approach. Regular reflection sessions among the facilitators assisted us to stay on course and for us to become more skilled at (and comfortable with) facilitation.

### From fixed courses towards increasing the options and providing resources

At the outset, the MPraxMed offered fixed courses. Steadily, we began to offer a range of options and resources to enable students to satisfy their learning needs, to become better doctors for their context.

Students were presented with a number of themes, which formed the content of the programme, such as Principles of Family Medicine, Whole Person Medicine, Ethics, the Family and Community, Culture, Research, among others. The themes had been presented as courses prescribed for particular year groups. Subsequently, students were provided with copies of core articles for each theme and lists of alternative references, advised regarding core and recommended books, and, increasingly, pointed to online resources. Students then decided, as individuals and in their learning groups, what they needed to address and in what way. This was pre-Google, so relied on a well-managed Resource Centre where students could find relevant material.

This ensured that instead of presenting information that was already known to students, or issues that were not relevant to their practices, they focused on what they needed to know at a particular time, and were thus much more open to learning – they could scratch *their* itches, not ours! This went beyond the flipped classroom concept, which came much later, but which has proven itself to be more effective than lecture-based learning.^19^

For new facilitators, this could be a scary process. It is much easier to prepare and ‘give’ a course! IC vividly remembers his first encounter with a newly enrolled group of students, who came expecting to be educated in the traditional way. When he threw the ball back into their court, he was not sure who was more anxious – the students as they tried to deal with this new concept of a lecturer who actually wanted to know what they needed to learn, or IC as he desperately hoped the students would come up with a topic that he knew something about!

### From speaking towards listening to the students’ agenda

To assist students to meet their learning needs, we first have to hear what these needs are. It is an interesting process to monitor the airtime in a group; in a traditional group, the leader or teacher will obviously dominate, whereas we were learning to take more and more of a backseat, to ensure the students had more airtime. It was a vital task to try to hear and understand our students and their needs in order to help them better.

This is, of course, directly analogous to a patient-centred consultation, and that is what we wanted to model. Fehrsen wanted our principles of teaching and learning to be congruous with our principles of consulting. So just as we aimed to be patient-centred, we aimed to be learner-centred, which implied actively listening to our students the way we actively listen to our patients. The importance of role-modelling in medical education is widely described in the literature, and is credited with integrating beliefs, values and principles into a doctor’s worldview, thus affecting professional identity formation.^20^ It is acknowledged that these effects depend on contextual, structural, cultural and organisational influences, including the relationship between students and preceptors.^20^ More significantly, it is argued that imitation may perpetuate undesirable practices, such as doctor-centred patient interviewing, which requires student-centred approaches to mitigate against this.^21^ There is scanty literature on the link between student centredness and patient centeredness, with only one study being found,^22^ but the parallels between learner–teacher and doctor–patient relationships have been observed,^23,24^ and we believe that there must be some carryover.

Obviously, we failed in this approach many times. We often felt we knew so much and desperately wanted to share all the information at our disposal, but as soon as we did that, we found that we stifled our students’ growth, just as we have seen happen when we dominate in our patient interactions.

### From posing as experts towards becoming co-learners

We recognised that we too were on a path of learning. Although we had additional experience and knowledge acquired over the years, these may not have been relevant or helpful to students. We were co-learners in a number of senses:

- We were still learning about family medicine and primary care in their broadest sense: we could not hope ever to master the entire body of knowledge that is required (and we needed students to understand that neither could they).
- We were still learning about rural health and the particular needs and demands of rural practitioners.
- We were still learning about learning and developing the programme: we saw this as an iterative and ongoing process, which required us to work with students as co-developers of the course.
- We were still learning about ourselves, with the belief that the more we knew about ourselves, the better able we would be to help others. Interacting with students helped us along that path.

In essence, once again we were modelling a way of being to our students that has implications for our relationship with our patients. Fehrsen’s philosophy was that as soon as we pose as experts, we not only set ourselves up for failure, but we might also reach a dead end where we stop learning, sit back and say we know enough.

In our early days as facilitators, we worried that students might ask us questions we could not answer, until we realised that it was putting ourselves back into the role of expert rather than co-learner.

### From being assessment-driven towards being relevant to students’ needs

The starting points of learning were the patients and the practice of each student. One of the key roles of the facilitator was to keep the students focused on their own patients and their own context. We believed that patients are the most valuable teachers, and that every patient interaction provides an opportunity for learning. Thus, patient interactions were what we sought to get our students to focus on.

Working within the structure of a university programme, assessment was required. Despite our best intentions to make the assessments relevant to practice, there was a tension that students could become more concerned about the examination than about improving their practice. We recognised that assessment can have unintended negative consequences for learning,^25^ although we did not at the time have the current language of assessment of learning versus assessment for learning.^26^.Intuitively we knew that we needed to consider the broader environment and culture in which assessment was taking place,^25^ and to try to ensure our assessments were driving the right kind of learning.^27^ We had introduced a form of more workplace-based formative and summative assessments, in that students were required to bring video recordings of real consultations from their clinical practices, which were very useful, despite technical challenges. We also started basing regional contact sessions near clinical facilities at which students worked, so that students could spend a few hours observing each other in consultations and offering feedback to their peers (an early form of workplace-based assessment), and we started practice visits to students’ own places of work, both as a mentorship and assessment tool. However, our assessment remained an element of incongruity in the course, which we struggled to resolve.

### From theoretical knowledge towards reflective action

Towards the end of the 1990s, we were trying to move students from acquiring theoretical knowledge to improving performance and practice through reflection. This was based on an understanding that the actions of an expert arise out of a complex integration of knowledge and skills that are appropriate to the particular context they are in and the situation that they face,^28^ and that reflection is the process of analysing and learning from one’s experiences to improve professional performance. This requires both reflection on action, which is reviewing a decision or event and considering all the factors that impacted the outcome, and reflection in action, being able to consider these same factors in real-time to modify a decision in order to improve an outcome.^29^ We began to build application of new knowledge and reflection on this action into all tasks and assignments. We were realising that knowledge is constructed through action and participation and were seeking to develop this theme further. We felt that if a student obtained the degree, but their practice was unchanged, then we had failed – the overarching aim was to strengthen care for patients.

During his own training, HC experienced a profound shift from being doctor-centred to becoming truly patient-centred. Patient-focused learning became real through his patient studies, each based on individuals he encountered in his everyday work. He still remembers many of these patients, not just their diagnoses, but their stories, their faces, and the lessons they taught him. This transformation also influenced his approach to teaching, making him more student-centred in his role as a facilitator of learning. His own lifelong learning, as well as the way he guides students in their learning journeys, continues to be deeply rooted in his patients.

### Implications

Although there was much less health professions education (HPE) literature available at the time, looking back now with an understanding of the evidence, we believe that the MPraxMed was already demonstrating a number of the approaches to learning that the evidence now urges educators to use,^30^ foreshadowing the role family medicine plays in driving change in medical curricula, both undergraduate and postgraduate, in South Africa and globally.^31^ An example is the development of a capability approach to learning.^32^

Postgraduate family medicine training in South Africa has continued to be at the forefront in implementing a number of these principles, including the critical importance of context, distributed learning and the value of patient centredness in training. This is not surprising given that at the time of Fehrsen’s death, five heads of the eight Departments of Family Medicine in the country were graduates from this programme.^1^

Facilitating learning by students who are empowered to regulate their own learning and for educators to be co-learners with our students requires reflection on and monitoring of our identity as educators. Flattening the traditional teacher-student hierarchy is still found to be threatening by many colleagues, especially because this approach does not always find acceptance among their teacher peers. At the same time, artificial intelligence is reinforcing the importance of moving from a focus on gaining knowledge to developing the ability to evaluate and use resources wisely. Assessment is moving strongly into the workplace. It is heartwarming to note the extent to which family physicians are at the forefront of driving workplace- and competency-based assessment in registrar training in South Africa. We urge our family physician colleagues to courageously continue to use and develop best educational practices with registrars in the hope that they too will develop not only patient-centred passion but also excellence in how they teach future generations.

### Reflexivity

The authors were part-time facilitators on the programme in the 1990s; three were also students on the programme preceding their role as facilitators (HC, JB and IC). We embrace our subjectivity as well as the unique insider insight that comes with that background. All authors have subsequently taught on other Family Medicine Masters programmes, have been engaged in education more broadly and have played leadership roles in Family Medicine in South Africa, all of which afford us unique perspectives from which to reflect on our experience.

## Conclusion

We offer these innovative principles for consideration as we all continue to think about our own learning and the design of the programmes with which we are involved. Family medicine should continue to innovate and drive change in medical training at both Postgraduate and Undergraduate levels. Family Medicine registrar programmes should be an exemplar for others to follow, taking note of the learnings in this article.

## Footnotes

* We have chosen deliberately to use the pronoun ‘we’ when referring to the team that facilitated the Medunsa MPraxMed programme, both because we were part of that team and because Sam Fehrsen’s leadership style was inclusive, so that all facilitators were part of the decision making for the programme.
